# Patient and caregiver experiences with hydrocortisone injections in adrenal crisis: a mixed-methods cross-sectional study

**DOI:** 10.3389/fendo.2025.1544502

**Published:** 2025-04-22

**Authors:** Sofia Llahana, Julia Anthony, Kyriakie Sarafoglou, Mitchell E. Geffner, Richard Ross

**Affiliations:** ^1^ School of Health and Medical Sciences, City St George’s, University of London, London, United Kingdom; ^2^ SOLUtion Medical, Philadelphia, PA, United States; ^3^ Divisions of Endocrinology and Genetics & Metabolism, Department of Pediatrics, University of Minnesota Medical School, Minneapolis, MN, United States; ^4^ Department of Experimental and Clinical Pharmacology, University of Minnesota College of Pharmacy, Minneapolis, MN, United States; ^5^ Department of Pediatrics, Division of Endocrinology, Diabetes, and Metabolism, Children’s Hospital Los Angeles, Los Angeles, CA, United States; ^6^ Clinical Medicine, School of Medicine and Population Health, University of Sheffield, Sheffield, United Kingdom

**Keywords:** adrenal insufficiency, adrenal crisis, parenteral glucocorticoid therapy, hydrocortisone injection, self-management, congenital adrenal hyperplasia

## Abstract

**Background:**

Adrenal crisis is the leading cause of death in patients with adrenal insufficiency, and prevention requires immediate parenteral hydrocortisone administration. However, most patients do not receive their home emergency hydrocortisone injection. Our study aimed to investigate barriers and enablers to using emergency hydrocortisone injections in managing adrenal crises.

**Methods:**

This mixed-methods observational study utilized an online survey distributed through two U.S.-based patient advocacy groups. A total of 688 respondents completed the survey, including 485 (70%) parents/caregivers of individuals with adrenal insufficiency and 203 (30%) adults with adrenal insufficiency. Qualitative free-text responses were analyzed using thematic content analysis, with subsequent quantification of identified barriers and enablers to administering parenteral hydrocortisone during adrenal crises.

**Results:**

Over 60% of patients with adrenal insufficiency had required parenteral hydrocortisone for an adrenal crisis, yet fewer than 20% managed to self-inject. Thirteen barriers and nine enablers were identified across three thematic domains: *device factors*, *external factors*, and *emotional factors*. Key barriers included the complexity of the multi-step hydrocortisone injection process (81%), injection-related anxiety and lack of confidence (18%), challenges accessing the correct hydrocortisone formulation or equipment (38%), and inadequate support for managing adrenal crises (29%). Key enablers included the effectiveness of hydrocortisone (14%), the convenience of the combined powder-and-diluent hydrocortisone vial (36%), and patient education (4%). Notably, 97% of participants expressed a preference for a hydrocortisone autoinjector to enhance self-injection capabilities.

**Conclusion:**

Effective adrenal crisis management requires comprehensive, evidence-based interventions across patient, healthcare, and societal levels. This should include the development of user-friendly hydrocortisone delivery devices, individualized patient education, healthcare system reforms, and public awareness.

## Introduction

Adrenal crisis is a life-threatening emergency characterized by circulatory collapse in patients with cortisol deficiency, often triggered by physiological stress, such as an infection (1). Each year, approximately 6 – 8% of patients with adrenal insufficiency experience a life-threatening adrenal crisis (2, 3). Prospective studies report an incidence rate of 8 adrenal crises per 100 patient-years (4, 5), while a retrospective study in the United States (US) found a higher rate of 24 per 100 patient-years, with 44% of patients having experienced at least one adrenal crisis since diagnosis (6). Mortality associated with adrenal crisis is 0.5 per 100 patient-years (4), indicating that 1 in 200 patients with adrenal insufficiency experiencing an adrenal crisis will die, despite its preventable nature (1). Gastroenteritis is the commonest precipitating factor for an adrenal crisis, although any physiological stressor that increases cortisol demand can precipitate a crisis (1, 2, 7).

There is no universally agreed-upon definition for adrenal crisis, though most accepted definitions emphasize acute deterioration and profound impairment of general health, with at least two of the following signs and symptoms: arterial hypotension with systolic blood pressure <100 mm Hg or relative hypotension (≥20 mm Hg lower than usual), nausea or vomiting, severe weakness or fatigue, fever, somnolence, confusion or impaired consciousness, hyponatremia (≤ 132 mmol/L), hyperkalemia, and/or hypoglycemia (more common in children), necessitating immediate parenteral glucocorticoids (1, 2, 7–12) resulting in a marked improvement in symptoms within two hours post parenteral glucocorticoid administration (2, 12).

The standard treatment for adrenal crisis is immediate parenteral hydrocortisone administration (2). In adults, the recommended initial dose is 100 mg of hydrocortisone given intramuscularly (ideally in the patient’s home setting) or intravenously, followed by 200 mg over 24 hours via continuous infusion or a 50-mg bolus every 6 hours in the hospital setting. In children, the initial bolus dose is 50 mg/m² of body-surface area, followed by 50 - 100 mg/m² over the next 24 hours (2, 8, 10, 11). Parenteral hydrocortisone is available as: 1) Hydrocortisone Sodium Phosphate 100 mg premixed liquid in an ampule (brand name Efcortesol^®^) and 2) Hydrocortisone Sodium Succinate 100 mg powder for solution (brand name Solu-Cortef^®^), either with separate diluent or combined as an Act-O-Vial^®^. Both formulations require approximately 15 steps to prepare and administer.

All patients/caregivers should be provided with an emergency hydrocortisone injection kit, an emergency care letter or steroid emergency card, and regular patient education, as timely administration of parenteral hydrocortisone can be lifesaving (9, 10, 13). In the case of mild or early symptoms of an impending adrenal crisis, parenteral hydrocortisone can prevent progression to a full crisis, and potentially the need for an emergency visit or hospitalization (2, 7, 13). Studies show that while over 70% of patients or caregivers possess and are trained to use hydrocortisone injection kits (14–17), fewer than 25% successfully self-inject or administer hydrocortisone during an adrenal crisis (15, 17–19), with rates as low as 12% in some cases (5, 18, 20). A recent US study reported that 41% of patients were unable to self-inject despite attempting to do so during an adrenal crisis (21). Additionally, only 20% of children with congenital adrenal hyperplasia received intramuscular hydrocortisone for stress dosing before presenting to the emergency department (19, 22).

Further significant delays in the administration of parenteral hydrocortisone by ambulance or emergency care personnel have often led to hospitalizations and intensive care admissions (14, 15, 19, 21, 23, 24). Research shows that immediate self-injection reduces hospitalization rates; 38% of patients self-injecting were hospitalized versus 73% who waited for a healthcare professional to administer their hydrocortisone (p=0.008), with 84% highlighting the complexity of the injection process as a barrier to self-injecting (25).

The hypotension and neurocognitive symptoms associated with an adrenal crisis often hinder a patient’s ability to self-inject, even if they are fully trained, thereby requiring assistance from a caregiver or healthcare professional (1, 2, 14, 26). The tragic case of an 11-year-old boy who died because ambulance personnel were not authorized to administer his personal hydrocortisone supply (11), highlights critical gaps in the current system, including the need for streamlined protocols and more user-friendly injection methods. In our study we investigated the barriers and enablers that patients with adrenal insufficiency and their caregivers report in using hydrocortisone injections for managing adrenal crises outside the hospital setting.

## Materials and methods

This observational study followed the STROBE Statement (27) and utilized the STROBE Checklist for Cross-sectional Studies to guide its design and reporting of the results. Patients with adrenal insufficiency and their caregivers, including parents, partners, and friends, were invited to participate in a cross-sectional online survey distributed through two patient advocacy organizations: CARES (Congenital Adrenal Hyperplasia Research, Education, and Support) Foundation (hereafter referred to as CARES) and Adrenal Insufficiency United (AIU). These organizations represent over 4,000 members, 85% U.S.-based, and a global reach across 70 countries. Recruitment methods included targeted emails, newsletters, and social media posts. A self-reported eligibility checklist determined if participants met the inclusion criteria, before proceeding with the survey.

### Inclusion criteria

- Adults older than 18 years of age diagnosed with primary, secondary, or tertiary adrenal insufficiency on glucocorticoid replacement therapy.
- Parents/caregivers of children or adults with primary, secondary, or tertiary adrenal insufficiency on glucocorticoid replacement therapy.

### Exclusion criteria

- Patients on supraphysiological doses of corticosteroids (not replacement therapy).
- Participants unfamiliar with hydrocortisone injections or adrenal crisis management.

The online survey was developed in collaboration with representatives from CARES and AIU, who contributed to content validation by assessing clarity, relevance, and ease of completion to ensure a neutral, patient-centered approach. It was designed using Google Forms^®^, and distributed by CARES (January-March 2019) and AIU (April-December 2019). The research team included an individual with lived experience of adrenal insufficiency (JA) and four clinicians with expertise in adult endocrinology (SL, RR), pediatric endocrinology (KS, MEG), and qualitative research (SL). The survey collected clinical and sociodemographic data, including adrenal insufficiency type, gender, age, and details of adrenal crises. While participants were not asked to specify when they or their child last experienced an adrenal crisis, they were instructed to base their responses on their most recent episode. As there is no universally accepted definition of adrenal crisis, we adopted a pragmatic approach, allowing participants to describe their experiences with administering hydrocortisone injections based on their own interpretation of an adrenal crisis rather than predefined criteria. Three open-ended questions invited participants to share their experiences with administering parenteral hydrocortisone during an adrenal crisis, highlighting helpful and frustrating aspects (enablers and barriers), and offering suggestions for improvement. The inclusion of open-ended questions and anonymized data collection allowed participants to provide unprompted responses, reducing the risk of investigator-imposed assumptions.

Quantitative data were analyzed using IBM SPSS V.28 Statistics for descriptive statistics and reported as mean, median, range, and standard deviation (SD) where applicable. Qualitative data from open-ended questions underwent inductive thematic analysis in NVivo^®^ version 13, with themes (barriers and enablers) subsequently quantified using SPSS. We applied Bengtsson’s four-stage Content Analysis Approach – decontextualization, recontextualization, categorization, and compilation – using manifest analysis to capture participants’ experiences with hydrocortisone injections (28). Blind coding of the qualitative data was conducted by SL and JA using an agreed coding guideline, to minimize bias in data interpretation. Reliability was assessed through theme concordance, with discussions to reach consensus. Data presentation and manuscript preparation underwent iterative review by KS, MEG, and RR to ensure accuracy and balance in reporting.

## Results

**Figure 1:**
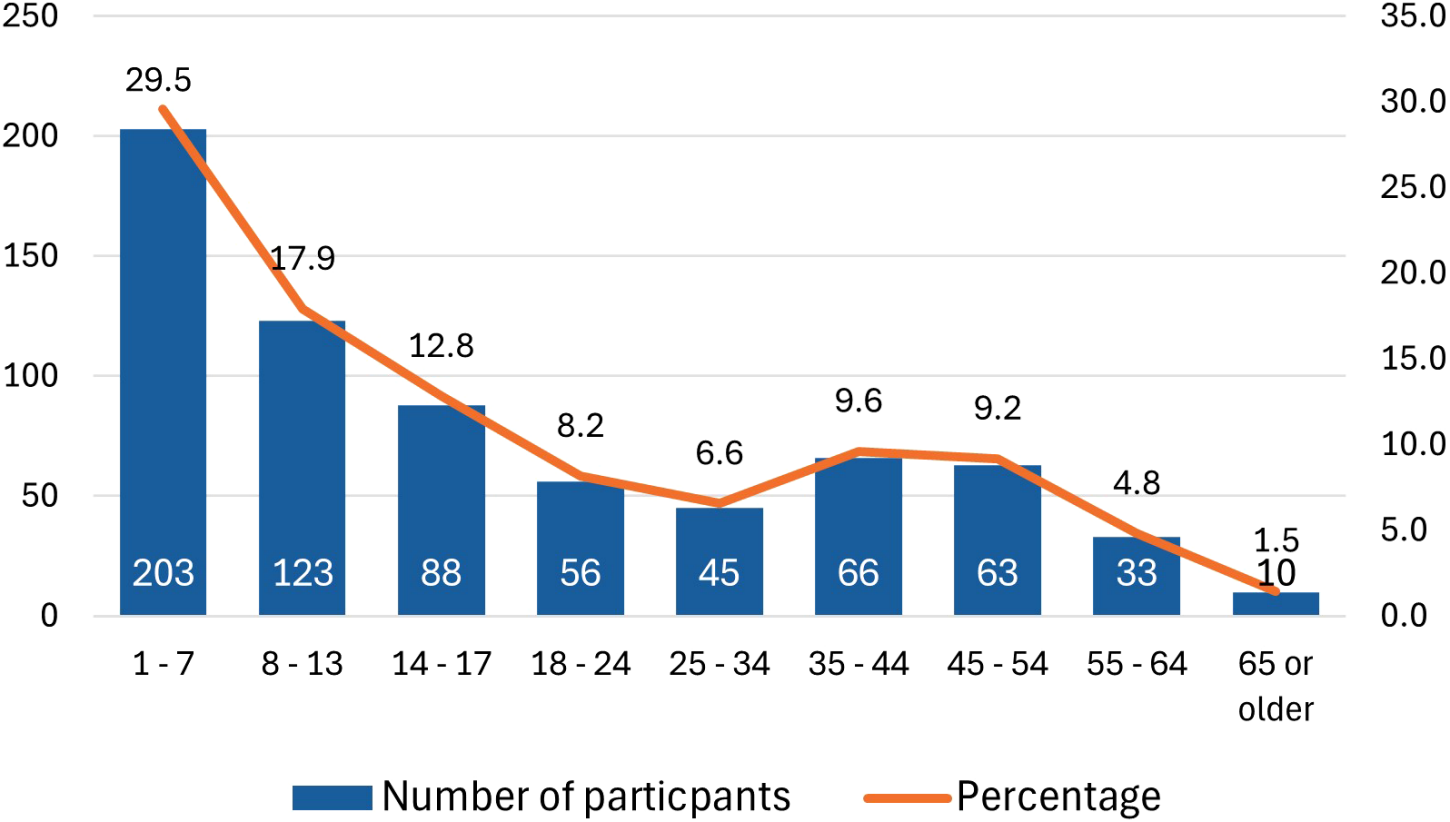
Age categories in children and adults with adrenal insufficiency (N = 687).

A total of 688 participants completed the survey: 248 (36%) from AIU and 440 (64%) from CARES (**Table 1**). The majority of participants were parents of children with adrenal insufficiency (N = 433; 62.9%), and over 80% of patients had primary adrenal insufficiency, which aligns with the main demographic served by these patient organizations. The mean patient age was 20.8 years (SD = 18.0), with 29.5% being children aged 7 years or younger (**Figure 1**). Most participants were U.S.-based and primarily used the Solu-Cortef Act-O-Vial^®^, referred to as the “hydrocortisone injection” in this study, unless specifically referencing the powder and vial (Solu-Cortef^®^) or the premixed (Efcortesol^®^) hydrocortisone formulation.

**Table 1:** Demographic and condition-specific characteristics (N = 688).

Demographic or condition characteristics	N (%)
Total number of participants	688
Parents/caregivers of children with AI	433 (62.9)
Adults with AI older than 18 years old	203 (29.5)
Parents, caregivers, partners, or other family members of adults with AI	52 (7.6)
Response ratio for CARES Foundation/AIU participants	440/248 (64/36)
Age (years)	687
Mean ± SD (standard deviation)	20.8 ± 18.0
Median (range min – max age)	14 (1 – 78)
Gender of patients with AI	688
Female	424 (61.6)
Male	244 (35.5)
Prefer not to say	20 (2.9)
Type of AI	686
Primary AI (including Addison’s Disease and CAH)	554 (80.8)
Secondary AI	114 (16.6)
Tertiary AI	5 (0.7)
Type of AI not known	13 (1.9)
Type of CAH in children with AI (CARES)	339/440 (77.0)
Classical Salt-Wasting CAH	269 (79.4)
Classical Simple-Virilizing CAH	35 (10.3)
Nonclassical or Late-Onset CAH	35 (10.3)
Type of CAH in adults with AI (CARES)	43/440 (9.8)
Classical Salt-Wasting CAH	26 (60.5)
Classical Simple-Virilizing CAH	6 (13.9)
Nonclassical or Late-Onset CAH	11 (25.6)

AI, Adrenal Insufficiency; CAH, Congenital Adrenal Hyperplasia; AIU, Adrenal Insufficiency United; CARES, Congenital Adrenal Hyperplasia Research, Education, and Support Foundation.

**Figure 2:**
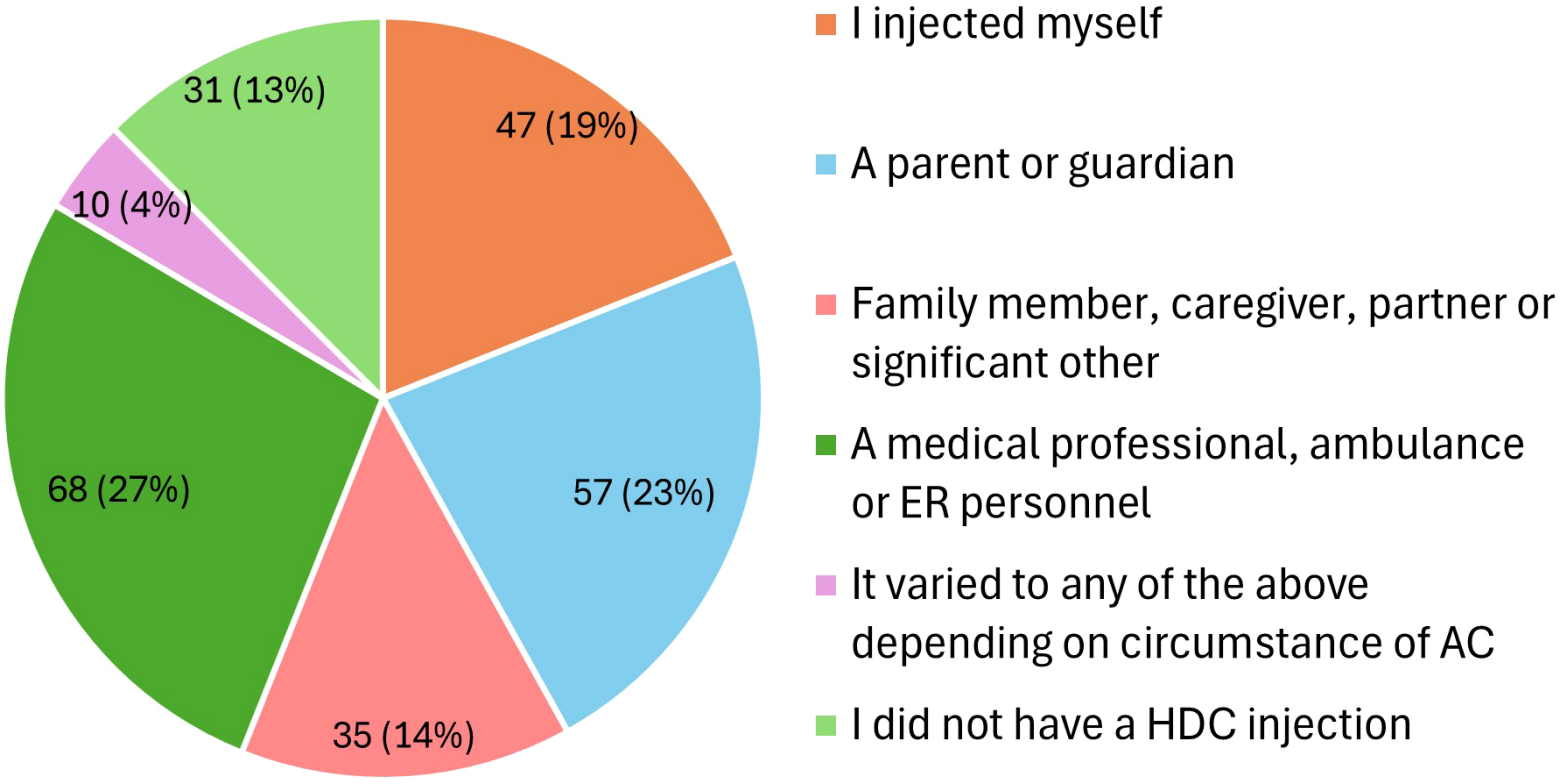
Who injected hydrocortisone in an adrenal crisis (N = 248; AIU respondents). AIU, Adrenal Insufficiency United patient organization; AC, adrenal crisis; HDC, hydrocortisone; ER, emergency room.

Among AIU participants, 87% (N = 215/248) required a hydrocortisone injection at some point to manage an adrenal crisis, but only 19% self-injected (**Figure 2**). In CARES group, where 81% of participants were parents of children with adrenal insufficiency, 63% (N = 277/440) reported having administered a hydrocortisone injection either to themselves or their child. The question for CARES participants differed slightly, focusing on whether participants had injected or if their child had self-injected. Among parents, only 14% (N = 60) indicated that their child had self-injected during an adrenal crisis.

**Figure 3:**
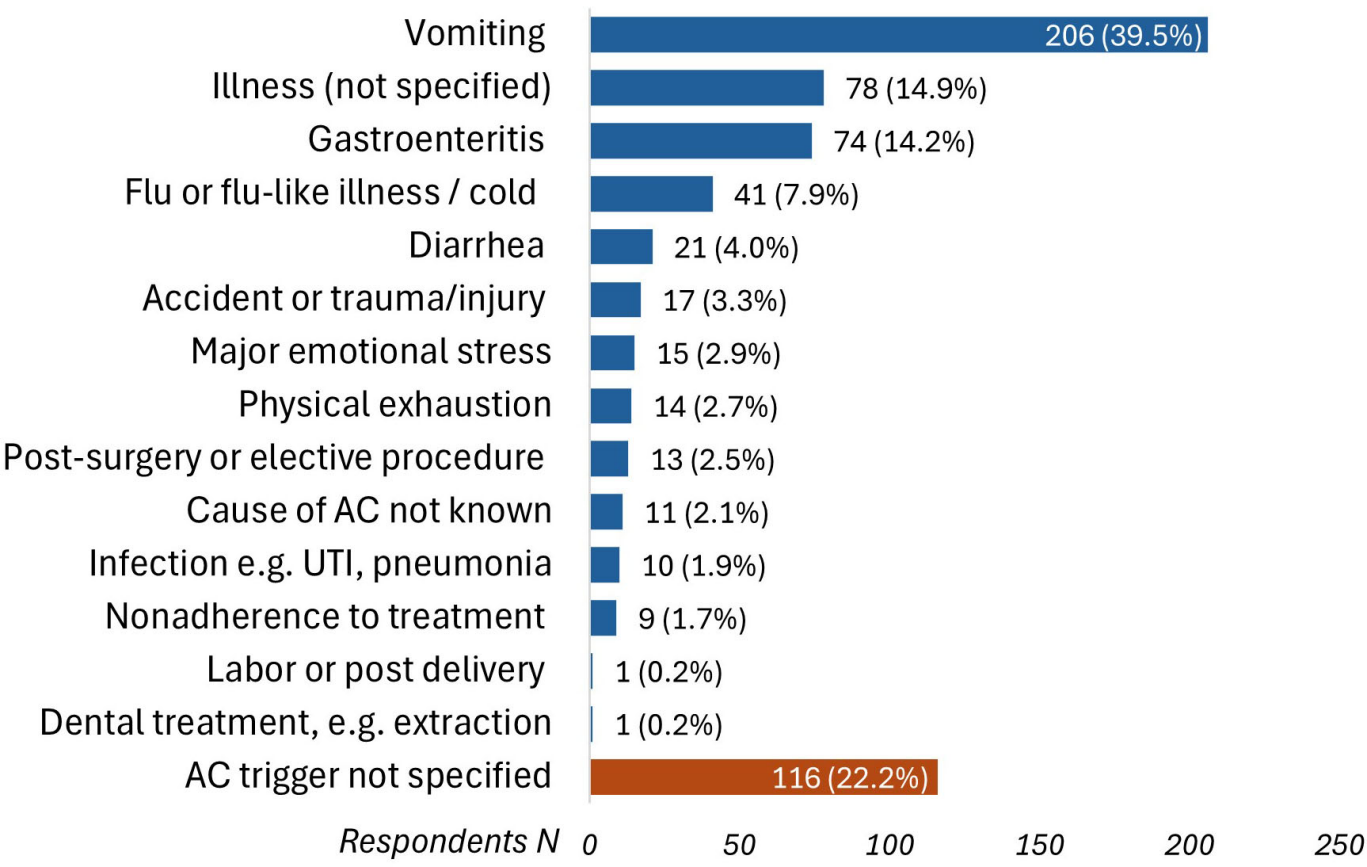
Precipitating circumstances that triggered adrenal crises (N = 522). AC, adrenal crisis; UTI, urinary tract infection.

Participants reported on circumstances surrounding their adrenal crises, with vomiting [39.5% (N = 206/522)] and gastroenteritis [14.2% (N = 74/522)] being the most common known precipitating factors. Of note, 22.2% could not identify any precipitating factors (**Figure 3**). Accessing hydrocortisone injections posed challenges for 38% (N = 262) of respondents, including pharmacies dispensing the wrong preparation (e.g., powder and saline instead of Act-O-Vial^®^) or failing to provide needles and syringes, which necessitated additional steps, such as returning to the pharmacy or obtaining new prescriptions, and in some cases, left participants unable to inject due to missing essential equipment.

**Figure 4:**
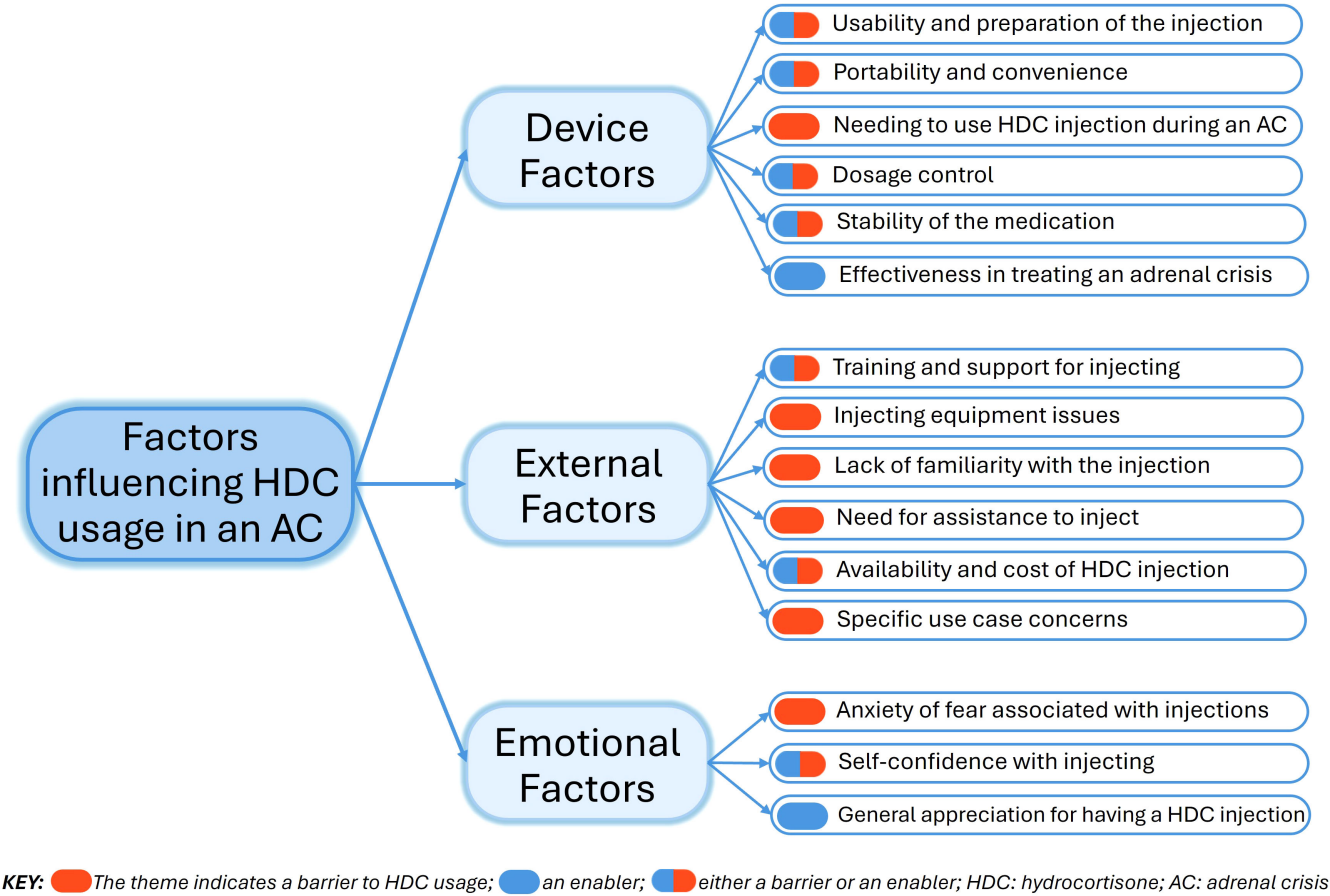
Domains and themes explaining barriers and enablers to hydrocortisone injection use during an adrenal crisis.

**Figure 5:**
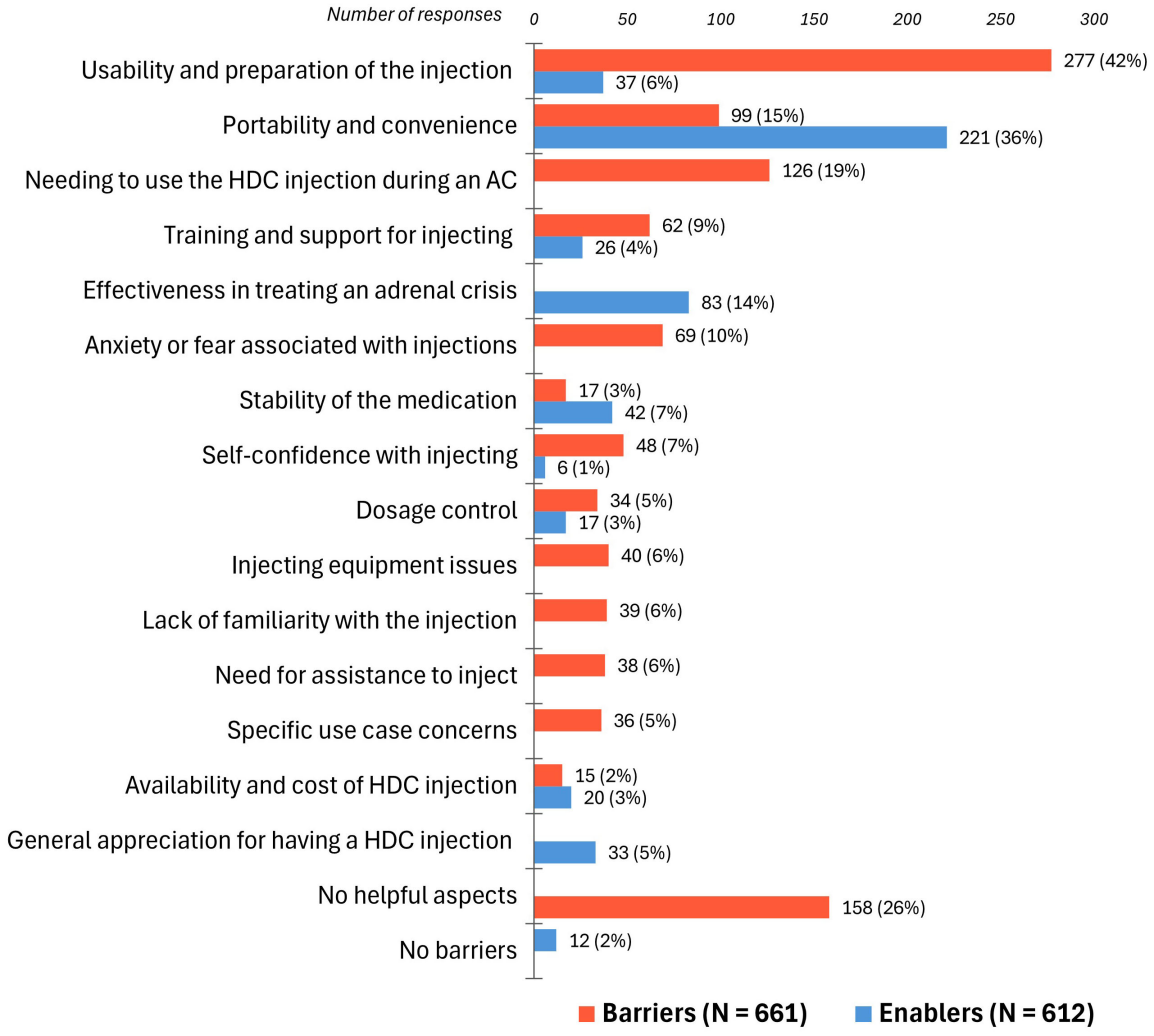
Barriers (N = 661) and enablers (N = 612) to hydrocortisone injection use during an adrenal crisis. HDC, hydrocortisone; AC, adrenal crisis. Participants often identified multiple barriers and enablers, with individual quotes coded into one or more themes. Bar labels represent the number and percentage of respondents attributing their experiences to each specific barrier or enabler.

The thematic analysis identified 15 themes (factors), grouped into three overarching domains: “Device Factors”, “External Factors”, and “Emotional Factors” (**Figure 4**). Barriers and enablers were quantified to assess their impact on participants’ experiences with using hydrocortisone injections for an adrenal crisis (**Figure 5**). Seven themes emerged as both barriers and enablers, depending on individual circumstances. Of the remaining eight themes, two were exclusively enablers and six were exclusively barriers. Notably, 26% (N = 158) of respondents reported finding “*no helpful aspects*” in the process of administering hydrocortisone injections during an adrenal crisis, while 2% (N = 12) reported encountering no barriers.

### Device factors

Usability and preparation of the injection was the most commonly reported barrier by 42% of respondents, who highlighted that the complex multi-step process often delayed the timely administration of parenteral hydrocortisone and caused anxiety over potential errors:

“*It is time consuming and difficult to complete in times of crisis. You are worried about your child and have to think through push down plunger, roll to mix, take off top, wipe with alcohol, take out needle, stick in vial, draw down, get out air, inject.*”

Six percent found the hydrocortisone injection process easy, typically after receiving regular injection training, if they were healthcare practitioners, or when not in an adrenal crisis:

“*Easy to do if I’m not sick and fading fast*”.

The portability and convenience of the Solu-Cortef Act-O-Vial^®^, appreciated for its all-in-one mixing design, was identified by 36% as an enabler:

“*The Act-O-Vial has everything in one, they tried to give me the other kind [Solu-Cortef^®^ powder and diluent] once and I panicked!*”

Barriers such as the fragile glass vial and bulky needles and syringes were reported by 15%:

“*Carrying needles and glass vials is something I’d prefer not to do. They are fragile and make me nervous with children digging through my purse*.”

Needing to inject hydrocortisone during an adrenal crisis was a significant barrier for 19% who faced challenges due to the complexity of the injection, worsened by the severely impaired health state during a crisis, as these respondents described:

“*It’s impossible to mix [the hydrocortisone vial] and change end of the needle while actively dying!*”

“*My hands and body shake so bad I break the needle, I can’t draw the medication up and my hands are too weak to activate [press down to mix] the vial*”

Respondents also described the impact that an adrenal crisis had on their family, and advocated for a simpler, more user-friendly injection device:

“*I am not calm in health crisis situations, so having to go through the whole process of preparing and giving my husband an injection is horrible. We are shocked that there isn’t an easier way … like an EpiPen. So many more lives would be saved!!!*”

Dosage control was cited as an enabler by 3% who valued the ease, accuracy, and reassurance of a premeasured hydrocortisone injection vial:

“*I know exactly how much I’m injecting*”.

Conversely, 5%, mainly parents of children with adrenal insufficiency, found measuring the correct dose in an adrenal crisis complex and stressful which acted as a barrier:

“*Having to remember what volume to inject for his age. At 6 years [of age] he still does not take the full vial (100 mg) in emergencies [adrenal crisis]*”

The stability of the medication was noted as an enabler by 7%. Conversely, 3% found this to be a barrier noting that hydrocortisone was not stable in extreme temperatures:

“*The sensitivity to heat makes it hard to take along all the time, as we have hot summers*.”

The effectiveness of hydrocortisone in treating an adrenal crisis was cited as an enabler by 14%, who highlighted its life-saving properties and rapid symptom relief despite the challenges of the complex injection process:

“*It keeps my son alive - but there’s nothing easy about it!*”

### External factors

Training and support for injecting were reported as an enabler by 4%. This included regular training and clear step-by-step instructions, provided through pictorial or video formats, which made the injection process less intimidating. In contrast, 9% reported that inadequate training and support, compounded by the complexity of the injection, acted as a barrier to managing adrenal crises. Many parents found it challenging to teach others, such as school staff, babysitters, and grandparents, who might need to inject their child in their absence. Further barriers included legal restrictions preventing school and daycare staff from administering hydrocortisone injections, often leading to inequalities in care:

“*Most non-medical professionals (schools, day area, etc.) refuse to do it, even though they DO agree to administer Epi-pens*”.

This concern also extended to some emergency care personnel:

“…*paramedics are also prohibited from administering patient-provided medications other than epi-pen style, and they do not carry Solu-Cortef*”.

Lack of familiarity with the injection was identified as a barrier by 6%, as caregivers, school staff, and some medical personnel felt intimidated by its complexity, leading to delays in administration. Parents expressed that this reluctance discouraged others from caring for their children, resulting in anxiety and isolation:

“*I constantly feel like my child’s life is placed solely in my hands and it affects me every time I leave him with someone unable to administer his shot*”.

Need for assistance to inject during an adrenal crisis was reported as a barrier by 6%:

“*I become incoherent and unable to move very quickly so I have to rely on someone else to inject me*”.

Many parents felt compelled to stay close to their children, adding logistical, financial, and emotional strain to the whole family:

“*It’s [the injection] a big source of anxiety for us, her [daughter], the school and her football coaches. She’s on tour abroad this year and I have to go with them and she has to travel with me not the squad. It impacts all our lives!*”

Specific use case concerns, such as attention-deficit/hyperactivity disorder, anxiety, visual impairments, dexterity problems, or tremor, were reported as a barrier and made the hydrocortisone injection especially challenging for 5%.

Availability and cost of hydrocortisone injections was an enabler for 3%, but a barrier for 2% due to insurance issues or difficulties obtaining the Act-O-Vial^®^ formulation. Four non-U.S. respondents reported that hydrocortisone injections were not available in their countries.

Injecting equipment issues, cited by 6%, were a barrier due to challenges in managing separate components like hydrocortisone, needles, syringes, diluent vials, and sharps boxes:

“*I need two prescriptions, one for saline and one for the powder [Solu-Cortef]*”.

### Emotional factors

Self-confidence with injecting was an enabler for only 1% of respondents, who felt confident using hydrocortisone injections, largely due to regular use for recurrent adrenal crises. In contrast, lack of self-confidence was a barrier for 7%, who attributed this to the complexity of the injection, compounded by the compromised health state:

“…*during an adrenal crisis I wouldn’t be clear headed enough to be able to go through all the steps*”.

Anxiety or fear associated with injections, cited as a barrier by 10%, resulted from the complex process of hydrocortisone administration and fear of dosage or technique errors:

“*An exposed needle that I have to properly inject under extreme stress and emotion [during a crisis]. Fear of doing it wrong. Fear someone else could do it wrong*”.

The reluctance of inexperienced caregivers also intensified respondents’ anxiety:

“*People refuse to accept my daughter in their care for fear of not being able to inject if necessary [in an adrenal crisis]*”.

General appreciation for having a hydrocortisone injection was cited as an enabler by 5%, with respondents expressing gratitude for its availability and highlighting global inequalities in access to this life-saving medication:

“*I’m glad to have hydrocortisone and grateful we don’t live in other places in the world where this may not be the case*”.

### Suggestions for improvement

Beyond the need for a simplified hydrocortisone injection device, respondents suggested enhanced training for healthcare providers, emergency services, and the general public; health insurance coverage; and legislating ambulance services to carry and administer hydrocortisone. When asked about their likelihood of using a hydrocortisone autoinjector instead of their current injection method, 93% (N = 637) responded “extremely likely,” 4% (N = 29) “likely,” 2% (N = 15) “maybe,” and only 1% (N = 7) responded “unlikely” or “extremely unlikely.”

## Discussion

To our knowledge, this is the first study to offer an in-depth understanding of the barriers and enablers associated with administering hydrocortisone injections during adrenal crises. Findings highlight the need for a holistic approach across the patient/caregiver, healthcare system, and society levels to address the challenges associated with the effective management of adrenal crisis using the current treatment approaches. The inclusion of both patients and caregivers (primarily parents) enabled a thorough exploration of the practical and emotional challenges of managing these high-stress, life-threatening situations, especially as caregivers often bear the responsibility of managing an adrenal crisis.

Vomiting was a precipitating factor for an adrenal crisis in 39.5% of patients in our study, consistent across adults and children, aligning with previous research (3, 18–20, 23). Other studies have reported gastrointestinal symptoms, including vomiting, as triggers for adrenal crises in more than 70% of patients with adrenal insufficiency (5, 17). In children, viral infections are more frequent triggers for adrenal crises compared to bacterial infections (12, 19, 29, 30). Emotional stress was reported as a trigger by 3% of patients in our study, consistent with earlier European studies (3, 18), but significantly lower than the 23% prevalence found in a U.S. study of 541 adult patients with primary adrenal insufficiency (23). This difference may reflect our predominantly pediatric patient population who are less likely to be exposed to or able recognize emotional stress. Recent guidelines from the United Kingdom recommend doubling or tripling the dose of oral glucocorticoids for 1-2 days during moderate to severe psychological stress (13), though this practice varies internationally.

Notably, 22.2% of participants in our study could not specify the precipitating factor for their adrenal crisis. This figure was lower than the 53% reported in a prospective study of children with CAH (29), but higher than the 6.6% and 12.7% in adults with primary and secondary adrenal insufficiency, respectively (3). The lack of a clear definition and low awareness of adrenal crisis may have contributed to participants’ difficulty in recognizing the onset of a crisis (2, 7), or may have led to the unnecessary administration of parenteral hydrocortisone when a crisis was not actually occurring. Furthermore, for many individuals with adrenal insufficiency, an adrenal crisis can occur without apparent triggers or symptoms, making it challenging to identify, especially in young children (12, 31). These findings highlight the need for a standardized definition of adrenal crisis and improved patient and caregiver education to enhance symptom recognition and ensure appropriate management.

### Device factors

Our study found that, while most patients at some point required a hydrocortisone injection to manage an adrenal crisis, less than 20% managed to self-inject. This aligns with previous studies showing self- or caregiver-administered injection success rates ranging between 12% and 24% (5, 15, 17–20), despite the fact that most patients possessed an emergency hydrocortisone kit and had been trained to use it (16, 25, 32, 33). However, no previous studies have provided an in-depth analysis of why patients and their caregivers struggle with administering hydrocortisone injections. One of the most significant barriers, identified by 42% of participants, was related to the complex multi-step process of the current hydrocortisone injection, which was more challenging in an adrenal crisis. These findings are consistent with prior research highlighting the difficulties that patients and caregivers face when administering hydrocortisone injections under duress (6, 16, 21, 25), with 84% of respondents indicating a need for a simplified user-friendly injection device such as an autoinjector (25). For instance, the outcome for the 11-year-old child described by Miller et al. may have been different with a hydrocortisone autoinjector, as his grandparents could have administered the injection and prevented the fatality (11).

### External factors

Our study found that patient education and training significantly impacted the success of hydrocortisone administration during an adrenal crisis. Evidence from standardized patient education programs shows improved knowledge and confidence with self-management (4, 33–35), as well as recognizing signs of an impending adrenal crisis (33). A recent study found a negative correlation between frequency of adrenal crises and the use of stress-dosing with oral hydrocortisone (r = −0.6, P <.001) (32), highlighting the importance of empowering patients and caregivers to take preventative action to avert crisis escalation. Annual participation in education programs has been recommended for patients with adrenal insufficiency and their caregivers (1, 7, 8, 10), as only 40% retain all the information delivered during any of these educational sessions (36). However, recent data show that 58% (N = 657) of patients and caregivers have never practiced an emergency injection in a training session (21). Furthermore, a study found that significantly fewer patients felt confident to self-inject 6–9 months after a 2-hour standardized education program compared to immediately after (p < 0.001), indicating a decline in competence and self-assurance over time (33), likely due to the complexity of the injection and the infrequent occurrence of adrenal crises for most individuals, underscoring the need for ongoing education. Education should be tailored to individual patient and caregiver needs, emphasizing adrenal crisis prevention, injection training, early recognition of symptoms, and addressing adrenal crisis-related anxiety and misconceptions (2, 7, 26, 37, 38). Only 14% of patients in our study had hydrocortisone administered by significant others (e.g., friends, family, school staff), attributing this to the complexity of the injection and legal restrictions in settings such as schools, where non-medical personnel are prohibited from administering injections.

Many participants in our study encountered healthcare professionals who refused to administer hydrocortisone, possibly due to the mistaken belief that glucocorticoid risks outweigh the dangers of withholding treatment (2). These findings align with previous evidence highlighting avoidable delays in adrenal crisis treatment (1, 11, 14, 15, 23, 25, 39). Surveys in America and Europe show that there are frequently long delays in providing emergency glucocorticoid treatment in the ambulance or emergency care settings (14, 15, 24). Several studies reported that 20–68% of patients/caregivers faced delays, even after alerting emergency personnel of life-threatening adrenal crises and presenting medical identification such as a bracelet or a steroid emergency card (6, 14, 23, 24, 40). This highlights the need for continued education (41–44), implementing alert systems and ambulance protocols (8, 45, 46), and heeding advice from well-informed patients and caregivers (1, 39). Standardized steroid emergency cards for children and adults have been successfully implemented across Europe and are available in more than 25 languages (9, 13, 47). Nearly 40% of participants reported difficulties obtaining essential injecting equipment and previous studies found that only 74% had a complete kit (17).

### Emotional factors

The emotional toll of managing adrenal crises was reflected in participants’ low self-confidence and anxiety about the complex injection process, even with prior training, due to fears of dosage errors, incorrect technique, or endangering their loved ones. Although patients and caregivers with experience of prior adrenal crises reported higher self-confidence in giving injections, a history of adrenal crisis increases the risk of further crises 3-fold (4) indicating that this is not always within the patient’s control. Self-confidence may also be hindered by perceived low necessity and concerns about glucocorticoid adverse effects (48, 49). Consistent with previous studies (6, 16, 18, 49), several participants found intramuscular hydrocortisone administration to be intimidating, leading to reluctance in injecting. As an alternative, subcutaneous administration of 100 mg hydrocortisone has demonstrated excellent pharmacokinetics for adrenal crisis treatment, with only an 11-minute delay in reaching target serum cortisol levels compared to intramuscular administration (50).

### Study strengths and limitations

The mixed-methods approach in this study enabled quantitative analysis from a large sample, providing scope for generalization, combined with in-depth qualitative insights into the factors influencing the administration of parenteral hydrocortisone during an adrenal crisis. Collaboration with patient advocacy groups ensured that the survey was relevant and appropriately targeted, thereby enhancing the validity of the findings. However, targeting participants with prior experience of adrenal crises may have introduced selection bias, as those without injection experience were excluded, potentially overlooking barriers related to initial training or access to treatment. Recruitment through patient advocacy groups may have further contributed to selection bias, as participants were likely to be more knowledgeable about their condition and engaged in self-management.

Additionally, the predominance of US-based participants, the high proportion of individuals with primary adrenal insufficiency (80%), and the relatively young mean age (20 years) may limit the generalizability of findings to the broader patient population. However, as supported by earlier studies, the identified barriers and enablers, particularly those related to injection complexity and confidence with self-injection during an adrenal crisis, are applicable across patients with different adrenal insufficiency types, age groups, and geographical regions (6, 16, 21, 25). Variability in adrenal crisis management has been reported despite standardized patient education (25), suggesting that these challenges extend beyond specific demographic groups. Another key limitation is that the survey did not include a standardized definition of adrenal crisis, leaving it open to participant interpretation. Additionally, participants were not asked to specify when their adrenal crisis occurred, potentially introducing variability in responses due to evolving management practices or recall accuracy. However, they were asked to consider their most recent crisis when responding to this survey.

### Clinical implications and recommendations

This study identified critical barriers to administering parenteral hydrocortisone during an adrenal crisis, highlighting the need for holistic interventions across patient/caregiver, healthcare system, and societal levels to address current gaps in patient care (**Table 2**). The need for easier-to-use, more intuitive injection devices, similar to autoinjectors used for anaphylaxis (51), is central to these interventions to minimize the cognitive and physical burden on patients and their caregivers and ensure timely treatment. However, as this study demonstrated, while a simplified injection device can positively impact user experience and promote timely treatment of adrenal crisis, it is not a standalone solution; comprehensive patient education, self-management strategies, and behavior change interventions grounded in evidence are essential for improving treatment adherence and health outcomes (38). Legislative changes, support for caregivers, healthcare professional training, education of non-medical personnel (e.g., school staff), and public awareness are also all critical.

**Table 2:** Gaps in the management of adrenal crisis with current parenteral hydrocortisone treatment and recommendations for improvement.

Level	Current gaps	Recommendations
Patient/Caregiver-Level	**Hydrocortisone device complexity:** Multi-step injection is challenging, especially in crises when physical and cognitive abilities are compromised, leading to life-saving treatment delays and heightened anxiety about injection errors, which impacts effective crisis response. **Inadequate patient education** affects patients’ and caregivers’ knowledge and confidence in managing adrenal crises, leading to delays and potentially preventable hospitalizations. **Lack of a universally agreed definition of adrenal crisis** creates diagnostic and management ambiguity, potentially leading to delayed treatment and negative patient outcomes.	**Simplified hydrocortisone device:** Develop a simple injection device, similar to autoinjectors used for anaphylaxis, to streamline administration, reduce errors, and increase confidence in managing adrenal crises, enabling timely self- or caregiver-administration to prevent avoidable hospitalizations and fatalities. **Individualized education:** Provide tailored education for patients and caregivers on crisis prevention, symptom recognition, and injection technique led by qualified professionals. **Establish a standardized adrenal crisis definition**, supported by targeted training for and universal diagnostic coding.
Healthcare System-Level	**Limited healthcare provider familiarity and perception gaps**: Insufficient training on adrenal crisis management results in delays or errors. Adrenal crisis is not universally viewed as a life-threatening emergency. **Resource constraints**: Limited resources restrict access to regular patient education and adequate provision of medication and injection equipment. **Ambulance and emergency response services** often lack supplies of hydrocortisone or authorization to administer patient-held medication during a crisis.	**Integrated training**: Include adrenal crisis management in medical and healthcare education curricula, shifting to a patient-centered culture respecting patient/caregiver insights. **Efficient Service Delivery**: Collaborate with patient advocacy groups for accessible education; a simpler device would make injection training easier, allowing more time for self-management and behavior change intervention. **Legislative reform**: Enable emergency personnel to carry and administer hydrocortisone at home; implement alert systems in medical records and standardized steroid emergency cards.
Society-Level	**Public awareness deficit**: Limited awareness of adrenal crisis and fear of injections due to their complexity hinder appropriate response, negatively affecting patients and families. **Support restrictions**: Legal barriers prevent non-medical personnel, such as school staff, from administering injections, resulting in avoidable hospitalizations. **Global Access Disparities**: Socioeconomic and geographic barriers often limit access to parenteral hydrocortisone for some patients with adrenal insufficiency, resulting in inequalities in care.	**Awareness campaigns**: Launch public education and lobbying initiatives on adrenal crisis management in partnership with patient advocacy groups, media, and community and political ambassadors. **Policy adjustments**: Permit trained non-medical personnel to administer injections in schools and public settings; an autoinjector similar to an epinephrine pen could alleviate fear and promote timely intervention. **Global access advocacy**: Collaborate with policymakers and manufacturers to ensure global access to hydrocortisone and simpler devices, addressing response disparities.

Overarching areas with identified gaps and strategies for improvement are indicated in bold font.

To assess the long-term impact of these findings, future research should evaluate progress across patient/caregiver, healthcare system, and societal levels. Follow-up mixed-methods and interventional studies can measure changes in self-injection rates, emergency response practices, and the adoption of technological advancements and simplified injection devices for adrenal crisis management. Prospective research involving diverse patient populations and healthcare professionals is also needed to assess the effectiveness of patient self-management initiatives, healthcare training, and legislative reforms. Monitoring these developments will provide critical insights to inform further improvements in patient care.

## Data Availability

The raw data supporting the conclusions of this article will be made available by the authors, without undue reservation.

## References

[1] Allolio B. Extensive expertise in endocrinology. Adrenal crisis. Eur J Endocrinol. (2015) 172:R115–24. doi: 10.1530/EJE-14-0824 25288693

[2] Rushworth RL Torpy DJ Falhammar H. Adrenal crisis. N Engl J Med. (2019) 381:852–61. doi: 10.1056/NEJMra1807486 31461595

[3] Hahner S Loeffler M Bleicken B Drechsler C Milovanovic D Fassnacht M. Epidemiology of adrenal crisis in chronic adrenal insufficiency: the need for new prevention strategies. Eur J Endocrinol. (2010) 162:597–602. doi: 10.1530/EJE-09-0884 19955259

[4] Hahner S Spinnler C Fassnacht M Burger-Stritt S Lang K Milovanovic D. High incidence of adrenal crisis in educated patients with chronic adrenal insufficiency: a prospective study. J Clin Endocrinol Metab. (2015) 100:407–16. doi: 10.1210/jc.2014-3191 25419882

[5] Tschaidse L Wimmer S Nowotny HF Auer MK Lottspeich C Dubinski I. Frequency of stress dosing and adrenal crisis in paediatric and adult patients with congenital adrenal hyperplasia: a prospective study. Eur J Endocrinol. (2024) 190:275–83. doi: 10.1093/ejendo/lvae023 38584334

[6] Li D Genere N Behnken E Xhikola M Abbondanza T Vaidya A. Determinants of self-reported health outcomes in adrenal insufficiency: a multisite survey study. J Clin Endocrinol Metab. (2021) 106:e1408–19. doi: 10.1210/clinem/dgaa668 PMC7947833 32995875

[7] Claessen KMJA Andela CD Biermasz NR Pereira AM. Clinical unmet needs in the treatment of adrenal crisis: importance of the patient’s perspective. Front Endocrinol (Lausanne). (2021) 12:701365. doi: 10.3389/fendo.2021.701365 34354671 PMC8329717

[8] Kienitz T Bechmann N Deutschbein T Hahner S Honegger J Kroiss M. Adrenal crisis—definition, prevention and treatment: results from a Delphi survey. Horm Metab Res. (2024) 56:10–5. doi: 10.1055/a-2130-1938 37562416

[9] Nowotny H Ahmed SF Bensing S Beun JG Brösamle M Chifu I. Therapy options for adrenal insufficiency and recommendations for the management of adrenal crisis. Endocrine. (2021) 71:586–94. doi: 10.1007/s12020-021-02649-6 PMC7929907 33661460

[10] Bornstein SR Allolio B Arlt W Barthel A Don-Wauchope A Hammer GD. Diagnosis and treatment of primary adrenal insufficiency: an Endocrine Society clinical practice guideline. J Clin Endocrinol Metab. (2016) 101:364–89. doi: 10.1210/jc.2015-1710 PMC4880116 26760044

[11] Miller BS Spencer SP Geffner ME Gourgari E Lahoti A Kamboj MK. Emergency management of adrenal insufficiency in children: advocating for treatment options in outpatient and field settings. J Investig Med. (2020) 68:16–25. doi: 10.1136/jim-2019-000999 PMC6996103 30819831

[12] Rushworth RL Torpy DJ Stratakis CA Falhammar H. Adrenal crises in children: perspectives and research directions. Horm Res Paediatr. (2018) 89:341–51. doi: 10.1159/000481660 29874655

[13] NICE. Adrenal insufficiency: identification and management. London: National Institute for Health and Care Excellence (2024). Available at: https://www.nice.org.uk/guidance/ng243. (Accessed October 15, 2025) 39631002

[14] Hahner S Hemmelmann N Quinkler M Beuschlein F Spinnler C Allolio B. Timelines in the management of adrenal crisis—targets, limits and reality. Clin Endocrinol (Oxf). (2015) 82:497–502. doi: 10.1111/cen.12609 25200922

[15] White KG. A retrospective analysis of adrenal crisis in steroid-dependent patients: causes, frequency and outcomes. BMC Endocr Disord. (2019) 19:129. doi: 10.1186/s12902-019-0459-z 31791297 PMC6889201

[16] Li D Brand S Hamidi O Westfall AA Suresh M Else T. Quality of life and its determinants in patients with adrenal insufficiency: a survey study at three centers in the United States. J Clin Endocrinol Metab. (2022) 107:e2851–61. doi: 10.1210/clinem/dgac175 PMC9202727 35350067

[17] Worth C Vyas A Banerjee I Lin W Jones J Stokes H. Acute illness and death in children with adrenal insufficiency. Front Endocrinol (Lausanne). (2021) 12:757566. doi: 10.3389/fendo.2021.757566 34721304 PMC8548653

[18] White K Arlt W. Adrenal crisis in treated Addison’s disease: a predictable but under-managed event. Eur J Endocrinol. (2010) 162:115–20. doi: 10.1530/EJE-09-0559 19776201

[19] Chrisp GL Maguire AM Quartararo M Falhammar H King BR Munns CF. Variations in the management of acute illness in children with congenital adrenal hyperplasia: an audit of three paediatric hospitals. Clin Endocrinol (Oxf). (2018) 89:577–85. doi: 10.1111/cen.13826 30086199

[20] Rushworth RL Gouvoussis N Goubar T Maguire A Munns CF Neville KA. Acute illness in children with secondary adrenal insufficiency. Clin Endocrinol (Oxf). (2021) 94:913–9. doi: 10.1111/cen.14427 33544418

[21] Hover WJ Krein AD Kallet J Kinney GL Speiser PW Witchel SF. People with adrenal insufficiency who are in adrenal crisis are frequently unable to self-administer rescue injections. Endocr Pract. (2025). doi: 10.1016/j.eprac.2025.02.017. In press. 40043845

[22] Tseng T Seagroves A Tanawattanacharoen VK Liang MC Koppin CM Keenan M. Electrolyte abnormalities and stress dosing predict illness-related hospitalizations among infants and toddlers with congenital adrenal hyperplasia. Clin Endocrinol (Oxf). (2023) 98:536–42. doi: 10.1111/cen.14876 PMC10006318 36593179

[23] Regan EA Vaidya A Margulies PL Make BJ Lowe KE Crapo JD. Primary adrenal insufficiency in the United States: diagnostic error and patient satisfaction with treatment. Diagnosis (Berl). (2019) 6:343–50. doi: 10.1515/dx-2019-0013 31256064

[24] Ibrahim C Mahon JL Fraser LA Van Uum S. Adrenal insufficiency, quality of life, and treatment in the emergency room. Endocr Rev. (2015) 36(Suppl_1). doi: 10.1093/edrv/36.supp.1

[25] Burger-Stritt S Kardonski P Pulzer A Meyer G Quinkler M Hahner S. Management of adrenal emergencies in educated patients with adrenal insufficiency—a prospective study. Clin Endocrinol (Oxf). (2018) 89:22–9. doi: 10.1111/cen.13608 29617051

[26] Llahana S Zopf K Mitchelhill I Grossman A. Prevention and management of adrenal crisis in children and adults. In: Llahana S Follin C Yedinak C Grossman A, editors. Advanced Practice in Endocrinology Nursing. Springer International Publishing, Cham (2019). p. 1183–205.

[27] von Elm E Altman DG Egger M Pocock SJ Gøtzsche PC Vandenbroucke JP. STROBE Initiative. Strengthening the reporting of observational studies in epidemiology (STROBE) statement: guidelines for reporting observational studies. BMJ. (2007) 335:806–8. doi: 10.1136/bmj.39335.541782.AD PMC2034723 17947786

[28] Bengtsson M. How to plan and perform a qualitative study using content analysis. Nurs Plus Open. (2016) 2:8–14. doi: 10.1016/j.npls.2016.01.001

[29] Ali SR Bryce J Haghpanahan H Lewsey JD Tan LE Atapattu N. Real-world estimates of adrenal insufficiency-related adverse events in children with congenital adrenal hyperplasia. J Clin Endocrinol Metab. (2021) 106:e192–203. doi: 10.1210/clinem/dgaa694 PMC7990061 32995889

[30] Tseretopoulou X Ali SR Bryce J Amin N Atapattu N Bachega TAS. Temporal trends in acute adrenal insufficiency events in children with congenital adrenal hyperplasia during 2019–2022. J Endocr Soc. (2024) 8:bvae145. doi: 10.1210/jendso/bvae145 39258010 PMC11387114

[31] Chrisp GL Torpy DJ Maguire AM Quartararo M Falhammar H King BR. The effect of patient-managed stress dosing on electrolytes and blood pressure in acute illness in children with adrenal insufficiency. Clin Endocrinol (Oxf). (2020) 93:97–103. doi: 10.1111/cen.14196 32301148

[32] Chifu I Burger-Stritt S Schrader A Herterich S Freytag J Kurlbaum M. Predisposing factors for adrenal crisis in chronic adrenal insufficiency: a case-control study. Eur J Endocrinol. (2023) 189:537–45. doi: 10.1093/ejendo/lvad149 38006230

[33] Burger-Stritt S Eff A Quinkler M Kienitz T Stamm B Willenberg HS. Standardised patient education in adrenal insufficiency: a prospective multi-centre evaluation. Eur J Endocrinol. (2020) 183:119–27. doi: 10.1530/EJE-20-0181 32580144

[34] Repping-Wuts HJ Stikkelbroeck NM Noordzij A Kerstens M Hermus AR. A glucocorticoid education group meeting: an effective strategy for improving self-management to prevent adrenal crisis. Eur J Endocrinol. (2013) 169:17–22. doi: 10.1530/EJE-12-1094 23636446

[35] van der Meij NT van Leeuwaarde RS Vervoort SC Zelissen PM. Self-management support in patients with adrenal insufficiency. Clin Endocrinol (Oxf). (2016) 85:652–9. doi: 10.1111/cen.13083 27063934

[36] Harsch IA Schuller A Hahn EG Hensen J. Cortisone replacement therapy in endocrine disorders—quality of self-care. J Eval Clin Pract. (2010) 16:492–8. doi: 10.1111/j.1365-2753.2009.01149.x 20210825

[37] Martel-Duguech L Poirier J Bourdeau I Lacroix A. Diagnosis and management of secondary adrenal crisis. Rev Endocr Metab Disord. (2024) 25:619–37. doi: 10.1007/s11154-024-09877-x 38411891

[38] Llahana S Mulligan K Hirani SP Wilson S Baldeweg SE Grossman A. Using the behaviour change wheel and person-based approach to develop a digital self-management intervention for patients with adrenal insufficiency: the Support AI study protocol. Front Endocrinol (Lausanne). (2023) 14:1207715. doi: 10.3389/fendo.2023.1207715 37455898 PMC10349524

[39] Wass JA Arlt W. How to avoid precipitating an acute adrenal crisis. BMJ. (2012) 345:e6333. doi: 10.1136/bmj.e6333 23048013

[40] Kampmeyer D Haas CS Moenig H Harbeck B. Self-management in adrenal insufficiency—towards a better understanding. Endocr J. (2017) 64:379–85. doi: 10.1507/endocrj.EJ16-0429 28190868

[41] Kampmeyer D Lehnert H Moenig H Haas CS Harbeck B. Experience pays off! Endocrine centres are essential in the care of patients with adrenal insufficiency. Eur J Intern Med. (2016) 35:e27–8. doi: 10.1016/j.ejim.2016.07.008 27444736

[42] Kampmeyer D Lehnert H Moenig H Haas CS Harbeck B. A strong need for improving the education of physicians on glucocorticoid replacement treatment in adrenal insufficiency: an interdisciplinary and multicentre evaluation. Eur J Intern Med. (2016) 33:e13–5. doi: 10.1016/j.ejim.2016.04.006 27108240

[43] Harbeck B Brede S Witt C Süfke S Lehnert H Haas C. Glucocorticoid replacement therapy in adrenal insufficiency—a challenge to physicians? Endocr J. (2015) 62:463–8. doi: 10.1507/endocrj.EJ14-0612 25739727

[44] Gaw AG Wemyss C Bell A Goodall CA. Management of patients at risk of adrenal crisis in the dental setting: a review of current practice in UK dental teaching hospitals. Br Dent J. (2022). doi: 10.1038/s41415-022-4515-0 35931749

[45] Mitchell AL Napier C Asam M Siddaramaiah N Heed A Morris M. Saving lives of in-patients with adrenal insufficiency: implementation of an alert scheme within the Newcastle-upon-Tyne Hospitals e-Prescribing platform. Clin Endocrinol (Oxf). (2014) 81:937–8. doi: 10.1111/cen.12457 24712680

[46] Mitchell AL Devine K Lal V Galloway P House M White K. Improving the prehospital safety of steroid-dependent patients in northern England: a hospital-initiated ambulance service registration pathway. Clin Endocrinol (Oxf). (2017) 87:881–2. doi: 10.1111/cen.13455 28834559

[47] Quinkler M Dahlqvist P Husebye ES Kämpe O. A European emergency card for adrenal insufficiency can save lives. Eur J Intern Med. (2015) 26:75–6. doi: 10.1016/j.ejim.2014.11.006 25498511

[48] Tiemensma J Andela CD Pereira AM Romijn JA Biermasz NR Kaptein AA. Patients with adrenal insufficiency hate their medication: concerns and stronger beliefs about the necessity of hydrocortisone intake are associated with more negative illness perceptions. J Clin Endocrinol Metab. (2014) 99:3668–76. doi: 10.1210/jc.2014-1527 25226291

[49] Chapman SC Llahana S Carroll P Horne R. Glucocorticoid therapy for adrenal insufficiency: nonadherence, concerns and dissatisfaction with information. Clin Endocrinol (Oxf). (2016) 84:664–71. doi: 10.1111/cen.12991 26641418

[50] Hahner S Burger-Stritt S Allolio B. Subcutaneous hydrocortisone administration for emergency use in adrenal insufficiency. Eur J Endocrinol. (2013) 169:147–54. doi: 10.1530/EJE-12-1057 23672956

[51] Vijayaraghavan R. Autoinjector device for rapid administration of drugs and antidotes in emergency situations and in mass casualty management. J Int Med Res. (2020) 48:300060520926019. doi: 10.1177/0300060520926019 32436421 PMC7243406

