# Supplementary material for: Patient and caregiver experiences with hydrocortisone injections in adrenal crisis: a mixed-methods cross-sectional study
Source: Front Endocrinol (Lausanne). 2025 Apr 22;16:1544502. doi: 10.3389/fendo.2025.1544502 (PMC12053486; doi:10.3389/fendo.2025.1544502)
Supplement: Supplementary file 1 [file Table1.docx]

**Supplementary Material – Survey questions**

*The survey was designed using Google Forms®, and distributed by CARES (January - March 2019) and AIU (April - December 2019); the survey included some additional question for the CARES Group, detailed below, however the open-ended and primary questions were the same across both surveys.*

**Do your responses to this survey come as a:**

*Mark only one oval.*

- Parent/Guardian to a child with adrenal insufficiency under age 18
- An adult with adrenal insufficiency
- A caregiver to an adult over 18 who has adrenal insufficiency
- Other: ________________________________________________

**What type of Adrenal Insufficiency does this person have?**

*Mark only one oval.*

- Primary
- Secondary
- Tertiary
- Unknown

**How old is this person?**

**________________ years**

**How old was this person when diagnosed?**

**________________ year**

**What is this person's gender?**

*Mark only one oval.*

- Female
- Male
- Prefer
- Other: _________________________

**Has this person needed an injection of solu-cortef?**

*Mark only one oval.*

- Yes
- No

**Who injected this person?**

*Mark only one oval.*

- Self
- A parent
- A different family member
- A friend
- A medical professional
- Other: _________________________

**How old was this person when the first injection was needed?**

**___________________ years**

**Can you please describe the circumstances surrounding the adrenal crisis event(s) and what triggered it?**

**What aspects of the current hydrocortisone medication injection process do you find helpful?**

**What aspects of the current Solu-Cortef Act-o-vial medication injection process do you find frustrating?**

**How would you like to see the process for administering Solu-cortef in adrenal crisis situations improved?**

**Has a pharmacy ever provided you with the incorrect medication for you / your child / anyone you know living with adrenal insufficiency? For example, pharmacies sometimes accidentally dispense powdered cortef to patients instead of the act-o-vial.**

*Mark only one oval.*

- Yes
- No
- I have received the powder but prefer it that way
- This is how it comes in my Country.

**Can you please describe this event?**

**How likely would you be to use a Solu-cortef auto-injector medical device?**

1 = never to 5 = extremely likely

***Additional questions asked by the CARES participants, data were stratified accordingly, or presented as a subset analysis.***

Are you a parent of a child with CAH?

*Mark only one oval.*

- Yes
- No

What type of CAH does your child have?

*Mark only one oval.*

- Classical Salt Wasting
- Classical Non Salt Wasting
- Nonclassical or Late Onset
- I do not have
- Other: __________________________

What is your child's age?

__________________________ years

What is your child's gender?

*Mark only one oval.*

- Female
- Male
- Prefer not to say
- Other: _____________________________

Has your child ever had to inject themselves with Solu-Cortef in an adrenal crisis?

*Mark only one oval.*

- Yes
- No

How old was your child when this occurred?

_____________________ years

Do you personally have CAH?

*Mark only one oval.*

- Yes
- No

What type of CAH do you have?

*Mark only one oval.*

- Classical Salt Wasting
- Classical Non Salt Wasting
- Nonclassical or Late Onset
- Other than
- Other: _________________________________

How old are you?

_______________________ years

What is your gender?

*Mark only one oval.*

- Female
- Male
- Prefer
- Other: ___________________________
